# Gsk-3-Mediated Proteasomal Degradation of ATF4 Is a Proapoptotic Mechanism in Mouse Pancreatic β-Cells

**DOI:** 10.3390/ijms232113586

**Published:** 2022-11-05

**Authors:** Yuko Nagao, Kikuko Amo-Shiinoki, Hiroko Nakabayashi, Masayuki Hatanaka, Manabu Kondo, Kimie Matsunaga, Masahiro Emoto, Shigeru Okuya, Yukio Tanizawa, Katsuya Tanabe

**Affiliations:** 1 Division of Endocrinology, Metabolism, Haematological Sciences and Therapeutics, Graduate School of Medicine, Yamaguchi University, Ube 755-8505, Japan; 2 Department of Diabetes Research, School of Medicine, Yamaguchi University, Ube 755-8505, Japan; 3 Health Administration Centre, Organisation for University Education, Yamaguchi University, Yamaguchi 753-8511, Japan

**Keywords:** apoptosis, endoplasmic reticulum stress, glycogen synthase kinase 3, activating transcription factor 4, pancreatic β-cell

## Abstract

Endoplasmic reticulum (ER) stress is a key pathogenic factor in type 1 and 2 diabetes. Glycogen synthase kinase 3 (Gsk-3) contributes to β-cell loss in mice. However, the mechanism by which Gsk-3 leads β-cell death remains unclear. ER stress was pharmacologically induced in mouse primary islets and insulinoma cells. We used insulinoma cells derived from Akita mice as a model of genetic ER stress. Gsk-3 activity was blocked by treating with Gsk-3 inhibitors or by introducing catalytically inactive Gsk-3β. Gsk-3 inhibition prevented proteasomal degradation of activating transcriptional factor 4 (ATF4) and alleviated apoptosis. We found that ATF4-S214 was phosphorylated by Gsk-3, and that this was required for a binding of ATF4 with βTrCP, which mediates polyubiquitination. The anti-apoptotic effect of Gsk-3 inhibition was attenuated by introducing DN-ATF4 or by knockdown of ATF4. Mechanistically, Gsk-3 inhibition modulated transcription targets of ATF4 and in turn facilitated dephosphorylation of eIF2α, altering the protein translational dynamism under ER stress. These observations were reproduced in the Akita mouse-derived cells. Thus, these results reveal the role of Gsk-3 in the regulation of the integrated stress response, and provide a rationale for inhibiting this enzyme to prevent β-cell death under ER stress conditions.

## 1. Introduction

The decline in functional β-cell mass is the central pathogenesis of type 1 and 2 diabetes [1,2]. Increased apoptosis plays a role in decreasing β-cell function and mass over time [3,4,5]. Owing to their high insulin secretory activity, β-cells are thought to be vulnerable to endoplasmic reticulum (ER) stress, a condition of disrupted ER homeostasis due to the accumulation of misfolded proteins [6]. ER stress is a key mediator of failing β-cells [7,8,9]. 

To adapt to ER stress, cells activate specific stress response mechanisms, such as the unfolded protein response (UPR) [10] and integrated stress response (ISR) [11], which are mediated by ER resident stress transducers. The former is mediated by inositol-requiring protein 1α (Ire1α) or activating transcription factor 6 (ATF6), while the latter is initiated by the activation of PKR-like ER kinase (PERK). PERK phosphorylates eukaryotic translation initiation factor-2α (eIF2α), resulting in an overall decrease in mRNA translation, but increased translation of select proteins, including activating transcription factor 4 (ATF4) [12,13]. ATF4 induces the transcription of genes involved in amino acid import, glutathione biosynthesis, protein synthesis, and resistance to oxidative stress [14,15]. Among the target genes, the growth arrest and DNA damage-inducible protein (GADD34) complexes with protein phosphatase 1 (PP1), and the holoenzyme complex GADD34: PP1 dephosphorylates eIF2α [16]. Instead, eukaryotic translation initiation factor 4E-binding protein 1 (4E-BP1) plays a key role in reprogramming protein translation via ATF4 [17]. Transcriptional regulation by ATF4 is necessary for the survival and identity of β-cells [17,18].

Glycogen synthase kinase 3 (Gsk-3) was originally identified as a serine/threonine kinase that inactivates glycogen synthase in the insulin signalling pathway [19]. Gsk-3, with two isoforms, Gsk-3α and Gsk-3β, is distinguished by the property of being constitutively active, whereas insulin/insulin like growth factor 1 (IGF-1) inhibit the kinase activity through PI3K/Akt-induced phosphorylation. In addition to inhibiting its activity, PI3K/Akt promotes nuclear export of Gsk-3β, thereby restricting its access to nuclear substrates [20]. Gsk-3 is implicated in a variety of cellular processes, including proliferation, migration, and metabolism, via the phosphorylation of various proteins, including transcription factors [21]. Gsk-3β has been implicated in β-cell failure in mouse models of diabetes [22,23]. Cellular models of ER stress and glucolipotoxicity show that attenuation of the Akt/Gsk-3 pathway is involved in β-cell apoptosis [24,25]. Here, we explored the role of Gsk-3 in the regulation of the ER stress response as a proapoptotic mechanism in mouse pancreatic β-cells.

## 2. Results

### 2.1. Gsk-3 Is Activated and Negatively Regulates ATF4 Expression under ER Stress

**Figure 1:**
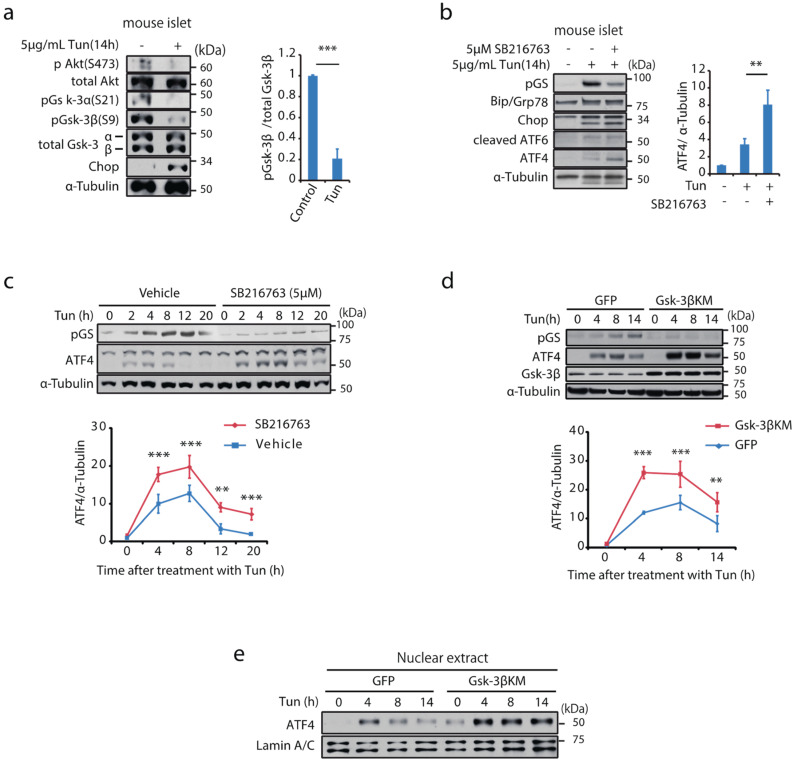
Gsk-3 negatively regulates ATF4 protein expression in mouse β cells under ER stress. (**a**) Isolated mouse islets were incubated with tunicamycin (5 μg/mL) for 14 h and Akt-Gsk-3 signalling was analysed by Western blot. Relative pGsk-3β/Gsk-3β are shown in the graph as mean ± SD (*n* = 3). *** *p* < 0.001 by unpaired student’s *t*-test. (**b**) Isolated islets were incubated with tunicamycin for 14 h in the presence or absence of GSK-3 inhibitor, SB216763 (5 μM) and protein lysates were analysed by Western blotting using indicated antibodies. Relative expressions of ATF4 are shown as mean ± SD (*n* = 3). ** *p* < 0.01 by one-way ANOVA followed by Bonferroni’s post hoc test. MIN6 in which Gsk-3 activity was inhibited by (**c**) SB216763 (5 μM) or (**d**) by retrovirus-mediated induction of HA-Gsk-3βKM were incubated with tunicamycin (5 μg/mL) for indicated periods. Protein lysates were analysed by Western blotting. Relative expression of ATF4 to the basal are expressed as mean ± SD (*n* = 3, respectively). ** *p* < 0.01, *** *p* < 0.001 by two-way ANOVA with Bonferroni’s post hoc test. (**e**) The nuclear extracts of MIN6 stably expressing GFP or HA-Gsk-3βKM were prepared after incubation with tunicamycin at indicated time points and were analysed by Western blotting using indicated antibodies. Representative images of two independent experiments are shown.

Impaired PI3K/Akt pathway by ER stress may lead to the deregulation of Gsk-3 activity in pancreatic β-cells [24,25,26]. To explore the changes in the Akt-Gsk-3 pathway under ER stress, mouse primary islets were incubated with tunicamycin to induce ER stress by inhibiting NH2-linked glycosylation. After a 14 h incubation, the inhibitory phosphorylation of Gsk-3α and Gsk-3β, was reduced along with a decrease in the phosphorylation of Akt-S473. There was >80% reduction in phosphorylation of Gsk-3β-S9 relative to the control (Figure 1a). Gsk-3 enzymatic activity, assessed by detecting phosphorylation of glycogen synthase (GS), was consistently elevated (Figure 1b). To assess the effects of Gsk-3 activity on unfolded protein response, islets were co-incubated with SB216763, a small chemical compound that selectively inhibits Gsk-3 kinase activity. The protein expression of Binding immunoglobulin protein/78-kDa glucose-regulated protein (Bip/Grp78), C/EBP homologous protein (Chop), cleaved ATF6, as well as the gene expression of Chop, Bip/Grp78 and spliced X-box binding protein 1 (Xbp-1) were not altered (Figure 1b and Supplementary Figure S1a). By contrast, there was more than a 2-fold increase in ATF4 protein expression in the presence of the Gsk-3 inhibitor. Time-dependent changes in ATF4 expression and the effects of Gsk-3 inhibition were examined in mouse insulinoma cells similarly incubated with tunicamycin. Although ATF4 protein expression was hardly detected at baseline, ATF4 expression induced by a treatment with tunicamycin was enhanced in the presence of the Gsk-3 inhibitor, and it was still detectable even after 12 h (Figure 1c). Consistent with the effects of the inhibitor, introducing Gsk-3βKM with a dominant negative effect on Gsk-3 activity [27] similarly enhanced ATF4 expression in whole cell lysates and in nuclear extracts (Figure 1d,e). Transcription and nascent protein synthesis of ATF4 were not altered by Gsk-3 inhibition, indicating that Gsk-3 activity might negatively regulate ATF4 through post-translational mechanisms (Supplementary Figure S1b).

### 2.2. Gsk-3 Negatively Regulates Protein Stability of ATF4

**Figure 2:**
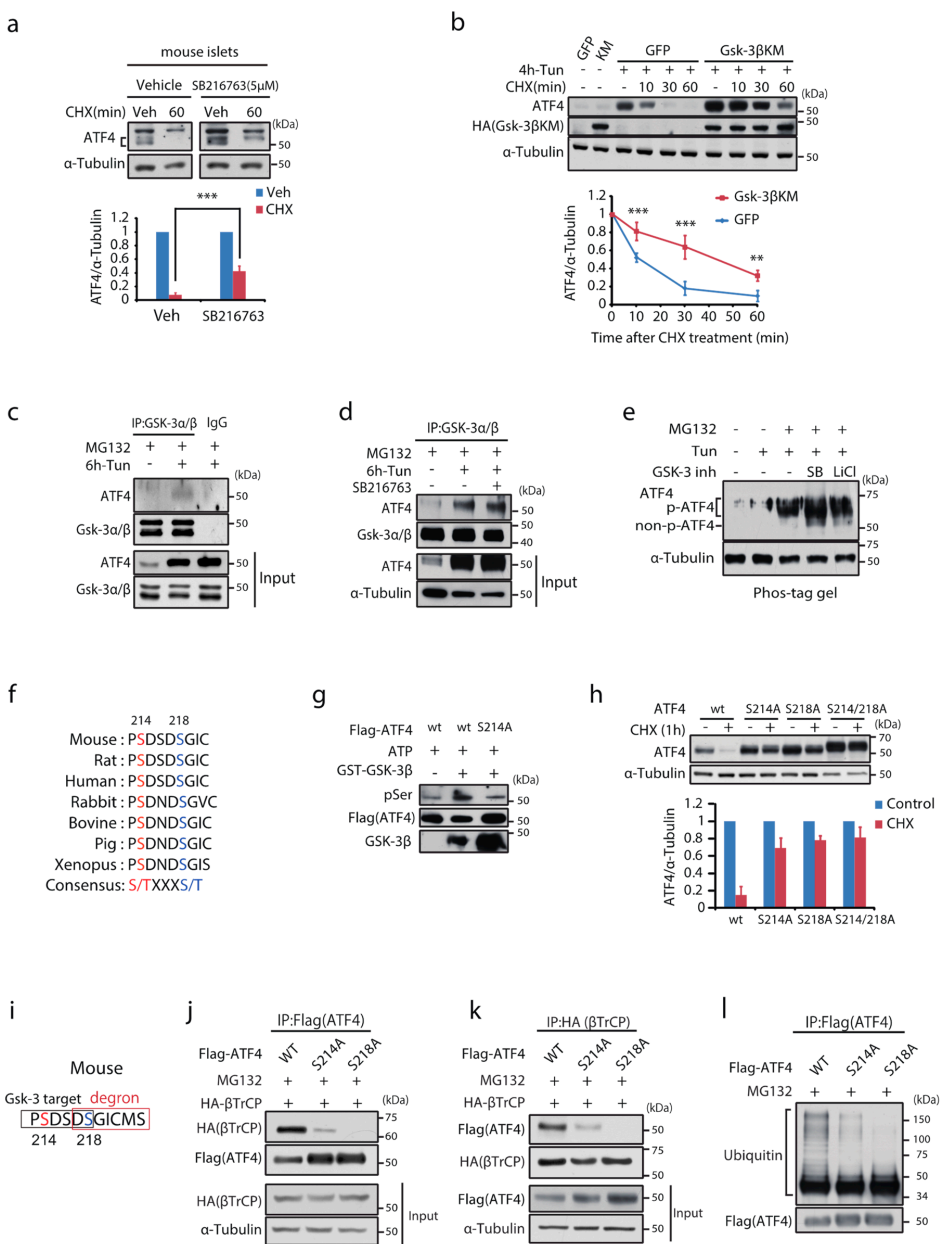
Gsk-3 promotes ATF4 protein degradation through phosphorylation of Ser 214. (**a**) CHX-chase assay for ATF4 protein in isolated islets incubated with tunicamycin (5 μg/mL) in the presence or absence of SB216763 (5 μM) for 4 h. Relative expression of ATF4 to the basal is shown as mean ± SD (*n* = 3). *** *p* < 0.001 by one-way ANOVA followed by Bonferroni’s post hoc test. (**b**) CHX-chase analysis for ATF4 induced by a 4 h incubation with tunicamycin in MIN6 stably expressing GFP or HA-Gsk-3βKM. Relative change of ATF4 to the basal of cells with GFP expression are expressed as mean ± SD (*n* = 3). ** *p* < 0.01, *** *p* < 0.001 by two-way ANOVA followed by Bonferroni’s post hoc test. MIN6 were incubated with tunicamycin (5 μg/mL) for 4 h, followed by a 2 h treatment with MG132 (25 μM). (**c**) Immunoprecipitation was performed using the anti-Gsk-3α/β antibody or non-specific IgG and the immunoprecipitants and the input were analysed by Western blotting using indicated antibodies. (**d**) MIN6 were similarly treated in the presence or absence of Gsk-3 inhibitor. The immunoprecipitants and the input were obtained using the anti-Gsk-3α/β antibody and were analysed by Western blotting. (**e**) Phos-tag analysis for ATF4 in MIN6 incubated with MG132 (25 μM) for 1 h following a 4 h incubation with tunicamycin in the presence or absence of Gsk-3 inhibitors. (**f**) Comparison of sequences of predicted Gsk-3 phosphorylation sites on ATF4 among various species. (**g**) In vitro GSK-3β kinase assay with wt and ATF4-S214A. (**h**) CHX-chase assay for ATF4 mutant series transiently expressed in HEK293. Change in expression is graphically shown as mean ± SD (*n* = 2). (**i**) The sequence of Gsk-3 target combined with the degron. HA-βTrCP was transiently expressed together with wt or ATF4 mutants (S214A and S218A) in HEK293. Following a 2 h incubation with MG132, protein interaction between βTrCP and ATF4 was examined in the immunoprecipitants obtained using (**j**) anti-Flag antibody for ATF4 and (**k**) anti-HA antibody for βTrCP. (**l**) The analysis of ubiquitination on wt and mutants of ATF4.

The role of Gsk-3 in protein stability of ATF4 was examined by pulse-chase analysis using cycloheximide (CHX). In mouse islets, ATF4 protein expression induced by ER stress rapidly declined after a CHX pulse, whereas it was significantly delayed in the presence of the Gsk-3 inhibitor (Figure 2a). The same effect of Gsk-3 inhibition was reproduced in MIN6 cells stably expressing Gsk-3βKM (Figure 2b). The decline in ATF4 after a CHX pulse was largely prevented in the presence of MG132, a proteasome inhibitor, indicating that ATF4 undergoes proteasomal degradation in mouse pancreatic β-cells (Supplemental Figure S2a). These observations suggest that Gsk-3 activity negatively regulates ATF4 protein stability through proteasomal mechanisms.

### 2.3. Gsk-3 Phosphorylates S214 of ATF4

We examined the physical interaction between Gsk-3 and ATF4. The pull-down assay performed on MIN6 cells incubated with tunicamycin demonstrated that endogenous Gsk-3 binds to ATF4 independently of its kinase activity (Figure 2c,d). Phosphorylation of ATF4 was examined by immunoblotting the protein samples dissolved in the Phos-tag acrylamide gel (Figure 2e). ATF4 induced by ER stress was detected at >50 kDa, an estimated molecular weight on SDS-PAGE, indicating that it was phosphorylated. Meanwhile, a major fraction, modestly smaller than the baseline, was detectable in the presence of MG132. When co-incubated with distinct types of Gsk-3 inhibitors, a subset of ATF4, presumably the non-phosphorylated form, appeared modestly >50 kDa. We identified a putative target of Gsk-3 in ATF4, which was confirmed to be conserved among various species (Figure 2f). Gsk-3 substrates require that a priming phosphate on the residue to be 4 amino acids C-terminal to the Ser/Thr being phosphorylated by Gsk-3 [21]. In this context, it has been demonstrated that Casein kinase phosphorylates S218 of ATF4 [28]. Therefore, we hypothesised that Gsk-3 might phosphorylate S214 of ATF4 following priming with phosphorylation on S218. An in vitro kinase assay using recombinant GSK-3β demonstrated that, whereas GSK-3β phosphorylated wt ATF4, this was inhibited in the S214A mutant (Figure 2g). Importantly, S214A and S218A both substantially increased the stability of ATF4 (Figure 2h).

### 2.4. Phosphorylation of S214 Is Required for Ubiquitination via the Degron of βTrCP

ATF4 is degraded via β-transducin repeat-containing protein (βTrCP)-mediated polyubiquitination [29]. Protein stability of ATF4 induced by ER stress was significantly increased in MIN6 cells stably expressing delta F βTrCP, a dominant negative mutant of βTrCP [29] (Supplementary Figure S2b). The same report also described that S218 is required for the βTrCP-mediated ubiquitination of ATF4. In addition, we found that the Gsk-3 target is conjugated with the degron of βTrCP and that S218 overlaps with both motifs (Figure 2i). As previously shown [29], the S218A mutation blunted the binding of ATF4 to βTrCP. Intriguingly, this interaction was largely disrupted by the S214A mutation (Figure 2j,k). Consistent with these findings, these mutants were resistant to polyubiquitination (Figure 2l). In addition to the mutation series, it was demonstrated that Gsk-3 activity was required for the binding of ATF4 to βTrCP and, in turn, its polyubiquitination (Supplementary Figure S2c,d). Conversely, ATF4 was downregulated by wortmannin, an inhibitor of PI3K, and the single amino acid substitutions S214A and S218A were resistant to wortmannin (Supplementary Figure S2e). Taken together, these findings illustrate that phosphorylation of S214 by Gsk-3 is required for the βTrCP-mediated proteasomal degradation of ATF4 (Supplementary Figure S2f).

### 2.5. Gsk-3-ATF4 Pathway Is a Proapoptotic Mechanism under ER Stress

**Figure 3:**
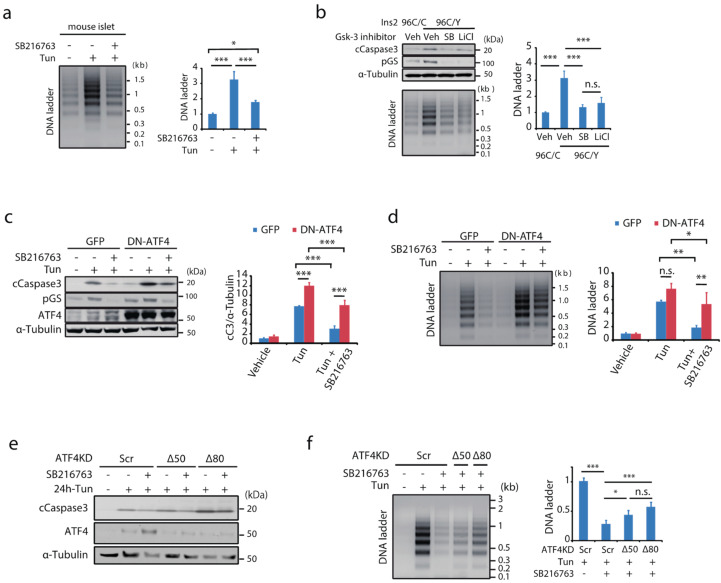
Inhibiting GSK-3 activity attenuates ER stress-induced apoptosis, dependently of ATF4. (**a**) DNA ladder assay in isolated islets incubated with tunicamycin for 20 h in the presence or absence of Gsk-3 inhibitor. Graphical data represent mean ± SD (*n* = 4). (**b**) Effects of Gsk-3 inhibition on serum deprivation-induced apoptosis in Ins2 96C/Y and Ins2 96C/C were assessed by detecting cleaved caspase 3 and DNA fragmentation. Graphical data are mean ± SD (*n* = 3). MIN6 stably expressing GFP or DN-ATF4 were incubated with tunicamycin for 20 h in the presence or absence of Gsk-3 inhibitor. (**c**) The protein lysates were analysed by Western blotting with indicated antibodies. Relative expression of cleaved caspase 3 is summarised in the graph (*n* = 3). (**d**) DNA ladder was examined. Relative amount of DNA ladder is expressed in the graph (*n* = 3). The effect of knock down of ATF4 (Δ50, Δ80) on anti-apoptotic action of Gsk-3 inhibition was examined in MIN6. ATF4 was knocked down by inducing shRNA targeting two distinct sequences of mouse ATF4. Following the ER stress treatment in the presence or absence of the Gsk-3 inhibitor, (**e**) expression of cleaved caspase 3 and (**f**) DNA ladder was examined. The graphs represent the relative abundance of the DNA ladder (*n* = 3). All statistical data were assessed by one-way ANOVA followed by Bonferroni’s post hoc test, * *p* < 0.05, ** *p* < 0.01, *** *p* < 0.001. n.s., not significant.

The involvement of Gsk-3 in ER stress-induced apoptosis was also examined. In isolated islets, tunicamycin treatment-induced apoptosis assessed by detecting DNA fragmentation was significantly suppressed by coincubation with the Gsk-3 inhibitor (Figure 3a). Consistent with the inhibitor, overexpression of Gsk-3βKM attenuated the induction of cleaved caspase 3 without affecting Chop expression as well as phosphorylation of Akt, upstream of Gsk-3 (Supplementary Figure S3a,b). In addition to pharmacological ER stress, in mouse insulinoma cells harbouring a heterozygous C96Y mutation, where Gsk-3 activity was elevated, distinct Gsk-3 inhibitors significantly decreased serum deprivation-induced apoptosis (Figure 3b). Conversely, introducing a dominant-negative form of ATF4 (DN-ATF4) [30], which lacks a transactivation domain, augmented ER stress-associated apoptosis, and the anti-apoptotic effect of Gsk-3 inhibition was largely attenuated (Figure 3c,d). In addition, two distinct shRNAs designed for knockdown of ATF4, which reduced ATF4 protein expression by 80% (Δ80) or 50% (Δ50) relative to the scramble shRNA, respectively (Supplementary Figure S3c), attenuated the anti-apoptotic action of Gsk-3 inhibition along with reducing ATF4 (Figure 3e,f).

### 2.6. Gsk-3 Modulates Integrated Stress Response

**Figure 4:**
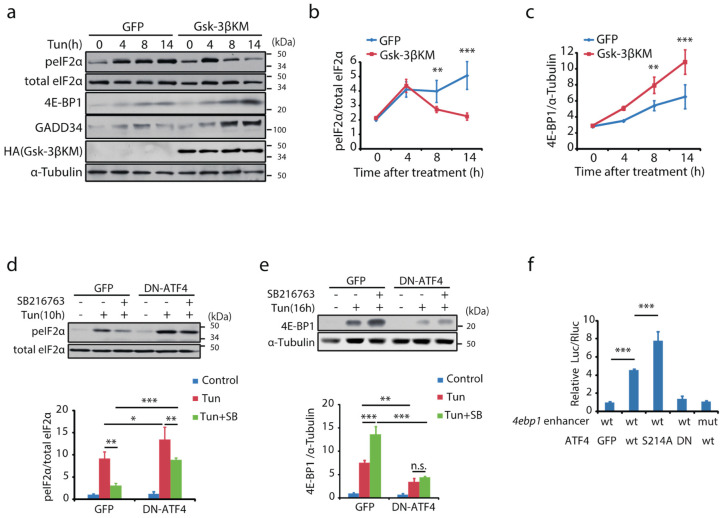
Gsk-3 modulates transcription targets of ATF4 and phosphorylation of eIF2α. MIN6 cells expressing GFP or HA-Gsk-3βKM were incubated with tunicamycin for the indicated time periods. Protein lysates were analysed by Western blotting. (**a**) Representative blot images obtained using the same gel. Representative changes in (**b**) peIF2αand (**c**) 4E-BP1 expression relative to the basal are graphically shown as mean ± SD (*n* = 3); ** *p* < 0.01, *** *p* < 0.001 by two-way ANOVA followed by Bonferroni’s post hoc test. The effects of retrovirus-mediated expression of DN-ATF4 on (**d**) peIF2α and (**e**) 4E-BP1 in MIN6 cells treated with Gsk-3 inhibitor were examined by Western blotting. Graphical data represent the relative abundance of peIF2α and 4E-BP1 as mean ± SD (*n* = 3). * *p* < 0.05, ** *p* < 0.01, *** *p* < 0.001 by one-way ANOVA followed by Bonferroni’s post hoc test. (**f**) MIN6 cells were transfected with luciferase reporters with the SV40 promoter and an Eif4ebp1 gene segment with C/EBP: ATF composite sites or their mutants (Supplementary Figure S5b) together with pcDNA3.1-GFP, pcDNA3.1-wt, -S214A, or -DN-ATF4. Luc/Rluc is graphically summarised. Data are shown as mean ± SD. *n* = 4 for each experiment; *** *p* < 0.001 by one-way ANOVA followed by Bonferroni’s post hoc test.

ATF4 is a key mediator of ISR during adaptation to ER stress [17,31,32]. The effects of Gsk-3 inhibition on the upstream and downstream regions of ATF4 were examined in MIN6 cells. eIF2α-S51 was similarly phosphorylated by a 4 h tunicamycin treatment, whereas it declined after 8 h in MIN6 cells where Gsk-3 was inhibited (Figure 4a,b and Supplementary Figure S4a). Simultaneously, the expression of GADD34, a transcriptional target of ATF4, was significantly increased, implying that dephosphorylation of eIF2α was facilitated (Figure 4a,b and Supplementary Figure S4a). In addition, the induction of 4E-BP1, another transcription target, was significantly enhanced by Gsk-3 inhibition (Figure 4a,c and Supplementary Figure S4a,b). The effects of Gsk-3 inhibition on eIF2α and 4E-BP1 expression were abolished by the introduction of DN-ATF4 (Figure 4d,e). Moreover, in isolated islets incubated with tunicamycin, the transcription of GADD34, 4ebp1, and Slc1a5 was confirmed to be potentiated by Gsk-3 inhibition (Supplementary Figure S5a). Gsk-3 in ATF4 transcription activity was examined in MIN6 transfected luciferase reporter plasmids carrying the Eif4ebp gene enhancer with C/EBP: ATF composite sites (Supplementary Figure S5b). The luciferase activity induced by tunicamycin treatment was significantly increased by the genetic or pharmacological inhibition of Gsk-3 (Supplementary Figure S5c,d). When co-transfected with plasmids expressing wt or mutated ATF4, ATF4-S214A was shown to be more potent in activating the reporter gene than wt (Figure 4f).

### 2.7. Gsk-3 Inhibition Modulates the Regulation of Protein Translation under ER Stress

**Figure 5:**
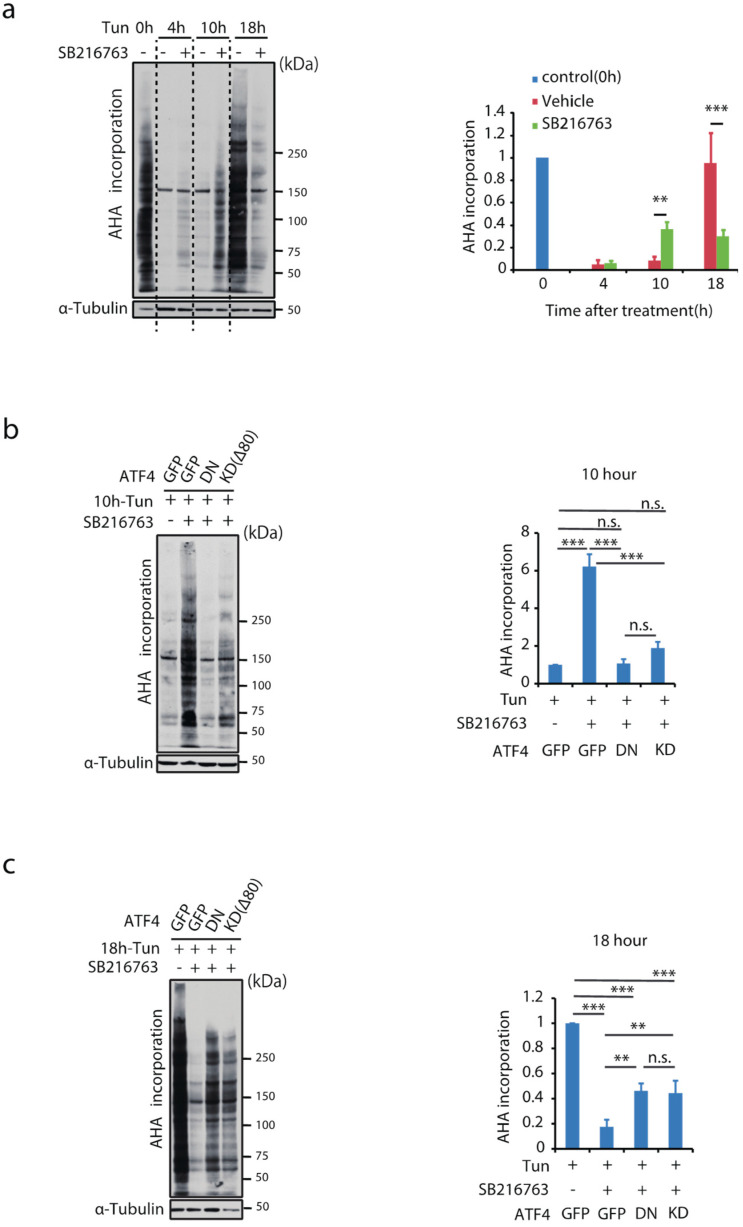
Inhibition of Gsk-3 activity alters change in global translation under ER stress. Metabolic labelling analysis using AHA in MIN6 cells treated with tunicamycin in the presence or absence of Gsk-3 inhibitor for the indicated time periods. AHA labelled nascent proteins in whole-cell lysates were assessed by Western blotting (**a**) using HRP-conjugated streptavidin (*n* = 3). Relative AHA incorporation corrected by α-tubulin was expressed graphically. ** *p* < 0.01, *** *p* < 0.001 by two-way ANOVA followed by Bonferroni’s post hoc test. Effect of inhibition of ATF4 function by introducing DN-ATF4 or shRNA(Δ80) on AHA incorporation following (**b**) a 10 h and (**c**) a 18 h treatment with tunicamycin (*n* = 3). Relative AHA incorporation corrected by α-tubulin is shown as mean ± SD in the graph. ** *p* < 0.01, *** *p* < 0.01 by one-way ANOVA followed by Bonferroni’s post hoc test. n.s., not significant.

We explored the functional relevance of ISR modulation by Gsk-3 inhibition. The effects of Gsk-3 inhibition on overall protein translation were assessed in MIN6 cells by incorporating AHA. As shown in Figure 5a, 4 h incubation with tunicamycin similarly suppressed protein translation in the presence or absence of the Gsk-3 inhibitor. At 10 h, protein translation was partially, but significantly, recovered by Gsk-3 inhibition. Conversely, reversal of protein translation at 18 h was disturbed by Gsk-3 inhibition. The involvement of ATF4 in the biphasic effect of Gsk-3 inhibition on protein translation was examined. Partial translational recovery by Gsk-3 inhibition at 10 h was blunted by introducing DN-ATF4 or KDΔ80 (Figure 5b), and translational suppression at 18 h was reversed by ATF4 inhibition (Figure 5c).

### 2.8. Gsk-3 Inhibition Potentiates ISR in Genetically Caused ER Stress

**Figure 6:**
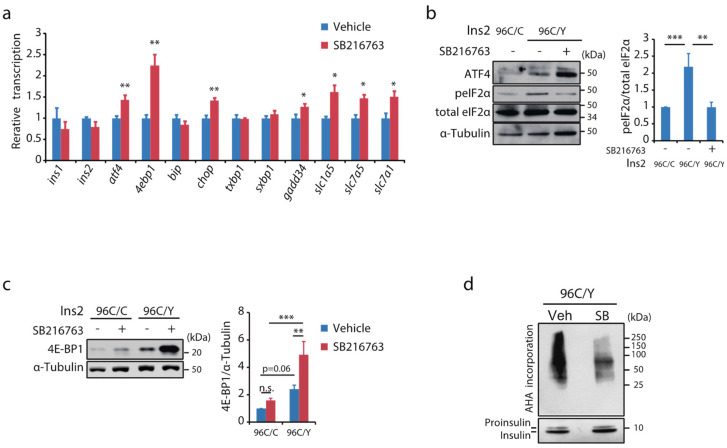
Gsk-3 inhibition modulates ISR in insulinoma cells carrying Ins2 mutation. (**a**) mRNA levels of indicated genes in the mouse insulinoma cells harbouring Ins2 96C/Y were assessed after a 16 h incubation with either GSK-3 inhibitor or vehicle (DMSO) (*n* = 3). The relative transcription levels were summarised as mean ± SD. * *p* < 0.05, ** *p* < 0.01 by unpaired Student’s *t*-test. (**b**,**c**) The protein extracts from cells with Ins2 96C/C or those with Ins2 96C/Y incubated with either Gsk-3 inhibitor or vehicle for 16 h were analysed by Western blotting using the indicated antibodies. Relative abundance of (**b**) peIF2α and (**c**) 4E-BP1 are summarised in the graph (*n* = 3, respectively). ** *p* < 0.01, *** *p* < 0.001 by one-way ANOVA followed by Bonferroni’s post hoc test. (**d**) Metabolic labelling analysis of cells with Ins2 96C/Y and those with Ins2 96C/C incubated with either vehicle or SB216763 for 20 h. The biotin-labelled proteins were collected using the streptavidin beads. The captured extracts were analysed by Western blotting using anti-biotin or anti-insulin antibodies.

**Figure 7:**
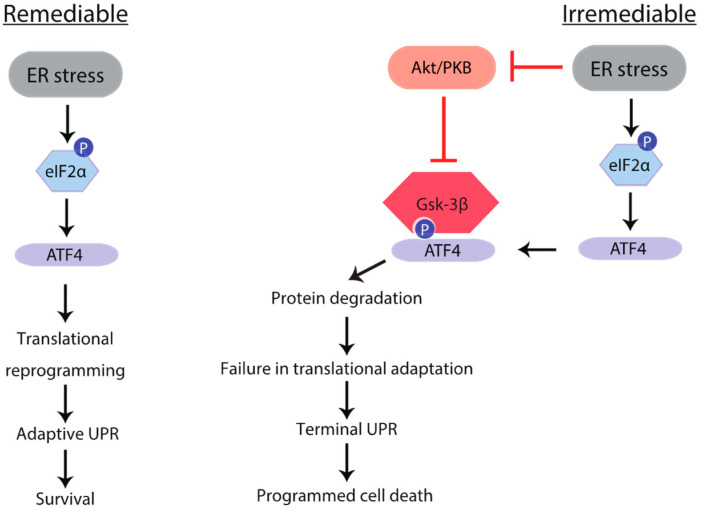
Schematic illustration of a role of GSK-3 on eIF2α-ATF4 pathway under ER stress. When β cells face ER stress, eIF2α phosphorylation suppresses global translation and paradoxically promotes ATF4 translation. ATF4 may switch translational suppression by eIF2α to more adaptive suppression by inducing 4E-BP1. In this context, attenuation of the Akt-GSK-3 pathway facilitates ATF4 protein degradation, and thereby ER stress becomes irremediable, leading to apoptosis.

Gsk-3 inhibitors attenuated apoptosis in insulinoma cells derived from Akita mice, a pathological model of ER stress-associated β-cell failure (Figure 3b). We examined the mechanisms underlying the anti-apoptotic effects of Gsk-3 inhibition. With chronic incubation with the Gsk-3 inhibitor, the transcription levels of 4ebp1, GADD34, and amino acid transporters, which are transcription targets of ATF4, were significantly increased (Figure 6a). Consistent with the observation of pharmacological ER stress, a chronic increase in ATF4 expression by Gsk-3 inhibition was associated with a decrease in phosphorylated eIF2α (Figure 6b) and an increase in 4E-BP1 expression without increasing inhibitory phosphorylation (Figure 6c and Supplementary Figure S6). Finally, the effects of Gsk-3 inhibition on protein translation were evaluated by metabolic labelling using AHA. Although global protein synthesis was strikingly suppressed, insulin biogenesis was not affected (Figure 6a,d), suggesting that Gsk-3 inhibition may alleviate the ER load without affecting insulin biogenesis in β-cells chronically exposed to ER stress. Taken together, the activation of Gsk-3 may negatively regulate ISR by facilitating ATF4 protein degradation, resulting in irremediable ER stress. Gsk-3 inhibition would make ER stress remediable, and hence may protect β-cells against ER stress-associated apoptosis (Figure 7).

## 3. Discussion

While the mechanism(s) that promote β-cell dysfunction in T2DM are still poorly understood, ER stress is thought to be a key mediator in this process [8,9,33]. Gsk-3, especially Gsk-3β, has proapoptotic properties in β-cells [22,24,25]. Although several biological substrates of Gsk-3 have been described, the roles of Gsk-3 in β-cell biology are yet to be fully understood. The current study explored the role of Gsk-3 in the regulation of ER stress response and cell death. We obtained the following novel findings: (1) ATF4 is a novel substrate of Gsk-3, (2) phosphorylation of S214 of ATF4 by Gsk-3 facilitates the binding of βTrCP to the degron, and (3) the Gsk-3-ATF4 pathway is a pro-apoptotic mechanism under ER stress. Hence, our findings demonstrate a novel crosstalk between growth factor signalling, such as insulin/IGF1, and ISR, and provide additional insights into the roles of Gsk-3 in pancreatic β-cells.

ATF4-S214 and -S218 consist of the consensus motif of Gsk-3. It has been previously reported that phosphorylation of ATF4-S218 by casein kinase is required for binding to βTrCP, which mediates polyubiquitination-proteasomal degradation [28,29]. Meanwhile, phosphorylation of S218 provides the priming phosphate for the phosphorylation of S214 by Gsk-3. In the current study, it was revealed that the consensus motif of Gsk-3 was conjugated with the degron, and that S218 was shared by both motifs (Figure 2i). In fact, we demonstrated that phosphorylation of S214 plays an essential role in the binding of βTrCP to the degron and subsequent polyubiquitination. Thus, proteasomal degradation of ATF4 is regulated by Gsk-3 activity and initiated by the phosphorylation of S214. Meanwhile, various phosphorylated Gsk-3 substrates are targeted to βTrCP, thereby leading to protein degradation [34]. Thus, the Gsk-3/βTrCP axis plays an important role in the regulation of various cellular functions under physiological and pathophysiological conditions. Our work adds ATF4 to a growing list of proteins regulated by the Gsk-3/βTrCP axis.

Gsk-3 activity is negatively regulated by the PI3K/Akt pathway. This could be blunted by ER stress. Attenuation of the Akt-Gsk-3β pathway is responsible for a high apoptotic cell rate, a low proliferation rate and a low insulin production in β-cells of *Irs2* deficient mice [22]. Given the results, Gsk-3β is more likely to be responsible for β-cell death and dysfunction than Gsk-3α. However, the tissue-specific role of each Gsk-3 isoform in pancreatic β-cells is yet to be elucidated and requires further study. Gsk-3β constantly shuttles between the nucleus and cytoplasm, even though it is predominantly a cytoplasmic protein [20]. Importantly, it has been described that Akt inhibits Gsk-3β to access nuclear substrates, and when Akt activity declines, Gsk-3β accumulates in the nucleus independently of its catalytic activity [20,35]. Therefore, when the Akt-Gsk-3β pathway is attenuated under ER stress, Gsk-3β can target ATF4 in the nucleus.

Gsk-3 modulates the integrated stress response mediated by eIF2α-ATF4. We showed that inhibition of Gsk-3 results in the upregulation of ATF4 transcription targets, 4E-BP1 and GADD34, and consequently dephosphorylates eIF2α without affecting its initial phosphorylation. Although global translational suppression by eIF2α phosphorylation due to PERK activation plays a role in the maintenance of β-cell homeostasis under ER stress, prolonged eIF2α phosphorylation may arrest cellular activities and consequently lead to cell death. Meanwhile, 4E-BP1 induction mediates another mode of translational control by eIF2α, and contributes to the maintenance of β-cell homeostasis during ER stress [17]. Since translational suppression by eIF2α phosphorylation is transient, owing to feedback dephosphorylation by GADD34 [36], prolonged translational suppression by 4E-BP1 might be required in the later stages of the UPR. These findings and the present results suggest that ATF4 functions to switch the translational control of eIF2α to that of 4E-BP1, a more adaptive mode. Thus, we speculate that when this process is prevented by Gsk-3, ER stress becomes irremediable, and cells fail to maintain homeostasis.

For a role of Gsk-3 under ER stress, the other potential pathways regulated by Gsk-3 need to be considered. Phosphorylation of eIF2Bε by Gsk-3 contributes to inhibition of eIF2 together with eIF2α phosphorylation [37,38]. This dual negative regulation of eIF2 potentially causes excessive translational suppression and a delay of translational recovery. Indeed, we demonstrated that Gsk-3 inhibition, although it also decreases eIF2α phosphorylation, results in an earlier reversal of translational suppression. Meanwhile, Gsk-3β reportedly plays a role in the regulation of autophagy under growth factor deprivation and ER stress conditions [39,40,41]. Gsk-3β phosphorylates HIV Tat-interactive protein, 60 kDa (TIP60), triggering a TIP60-mediated acetylation of Unc-51-like kinase 1 (ULK1) and activation of autophagy. Interestingly, the attenuation of Gsk-3β-TIP60-ULK1 pathway renders HeLa cells more sensitive to and increases the toxicity of ER stress [41]. This adaptive cytoprotective action of Gsk-3β is opposite to the action in β-cells. Therefore, a role of Gsk-3β under ER stress might be different in cell types. Further studies will be needed to explore a role of Gsk-3-mediated autophagy in β-cells under ER stress.

While cleaved caspase 3 and DNA ladder were tested, the current study lacks detection and quantification of individual dead or apoptotic cell. This raises a potential limitation on evaluation of β-cell death and apoptosis. Moreover, the in vivo relevance of the findings of our study remains unknown. Further studies are needed to explore the in vivo role of Gsk-3 activity under ER stress conditions. This is also a limitation of this study.

## 4. Materials and Methods

### 4.1. Cell Culture and Reagents

The mouse insulinoma cell line MIN6 (passage 22–32) [42], and insulinoma cells derived from the Akita mouse and from normal littermates, Ins2 96C/Y cells and Ins2 C/C cells [43], were grown in monolayer cultures. These cells were maintained in Dulbecco’s modified Eagle’s medium (DMEM) (Sigma-Aldrich, St. Louis, MO, USA) supplemented with 15% foetal bovine serum and penicillin/streptomycin (100 units/mL and 50 μg/mL, respectively) in an atmosphere of 5% CO_2_ at 37 °C. The genotype for the insulin-2 gene was confirmed by restriction fragment length polymorphism (RFLP) as previously described [44]. To examine serum deprivation-induced apoptosis, Ins2 96C/Y and Ins2 C/C cells were cultured in serum-free media for 24 h. HEK293 cells were purchased from ATCC (CRL-1573) and grown in DMEM supplemented with l-glutamine, 10% foetal calf serum. Tunicamycin, thapsigargin, and cycloheximide were purchased from Sigma-Aldrich. SB216763 was purchased from Tocris Bioscience, and MG132 was from CALBIOCHEM (San Diego, CA, USA).

### 4.2. Islet Isolation

Islets from 12-week C57BL/6J male mice were isolated by ductal collagenase distension/digestion of the pancreas [45] followed by filtering and washing through a 70 µm nylon cell strainer (BD Biosciences, San Jose, CA, USA). Isolated islets were then maintained in RPMI medium containing 11 mM glucose, 10% FBS, 200 units/mL penicillin, and 50 μg/mL streptomycin in humidified 5% CO_2_, 95% air at 37 °C. All experiments on isolated islets were performed after 24 h of culture following isolation. Isolated islets were treated with 5 μg/mL tunicamycin in the presence or absence of a Gsk-3 inhibitor (5 μM for SB216763, 10 mM for LiCl) for 14–20 h.

### 4.3. Plasmids

Full-length murine Gsk-3β and ATF4 were cloned by RT-PCR amplification using total RNA from MIN6. Human βTrCP1 cDNA was obtained from DNAFORM (Yokohama, Japan). Single and double mutations in ATF4 or Gsk-3β were constructed using PCR-based site-directed mutagenesis. WT and mutant cDNAs tagged with Flag (for ATF4) or HA (for Gsk-3β, βTrCP1) were subcloned into pcDNA3.1 (Invitrogen, Carlsbad, CA, USA). A catalytically inactive Gsk-3β (Gsk-3β-KM), in which lysine residues at positions 85 and 86 are mutated to methionine and alanine, respectively [27]. A dominant negative mutant of ATF4 (DN-ATF4) was generated by mutagenesis using PCR, resulting in a protein with 6 amino acid substitutions within its DNA-binding domain (292RYRQKKR298 to 292GYLEAAA298) [30]. We constructed a dominant-negative mutant of βTrCP1 lacking the F-box (delta F βTrCP), as previously described [46].

### 4.4. Retrovirus Vector and shRNA of ATF4

HA-Gsk-3βKM, Flag-DN-ATF4, and HA-delta F-βTrCP were sub cloned into the retroviral vector pMXs-puro (Cell Biolabs, San Diego, CA, USA). Two sequences used for the retroviral-mediated shRNA expression vector (pSirenQ; Invitrogen) targeting ATF4 were 5′-AAGCCTGACTCTGCTGCTTAC-3′ and 5′-AACCATGCCAGATGAGCTC-3′. Synthetic complementary single-stranded oligonucleotide DNA (Supplementary Table S1) was annealed to generate double-stranded DNAs of the target sequences. Annealed DNA was then inserted into the vector. The retroviruses were produced in HEK293T cells. MIN6 cells were transduced for 4 h with the virus and polybrene (Sigma-Aldrich) at 8 μg/mL. Following infection of MIN6 cells with the retrovirus, cells were selected by incubation with puromycin (2 days, 0.5 μg/mL). The cells were allowed to recover for 3 days before collection or stress treatment. 

### 4.5. Cycloheximide-Chase Assay

Isolated mouse islets were treated with tunicamycin for 4 h with or without GSK-3 inhibition, and were then incubated with 40 μM cycloheximide (CHX) for 60 min. MIN6 cells were similarly treated and harvested 10, 30, and 60 min after the addition of CHX. HEK293 cells transfected with pcDNA3.1 carrying wt or ATF4 mutants were incubated with CHX for 60 min. Following CHX-chase treatment, protein samples were prepared and analysed by Western blotting.

### 4.6. Western Blotting

Insulinoma cells or mouse islets were washed twice in ice-cold phosphate-buffered saline and lysed in ice-cold cell lysis buffer consisting of 50 mM HEPES (pH 7.5), 1% (*v*/*v*) Nonidet P-40, 2 mM activated sodium orthovanadate, 100 mM sodium fluoride, 10 mM sodium pyrophosphate, 4 mM EDTA, 1 mM phenylmethylsulfonyl fluoride, 1 µg/mL leupeptin, and 1 µg/mL aprotinin, then passed 10 times through a syringe with a 29-gauge needle. The particulate material from both cell lines was removed by centrifugation (10,000× *g*; 10 min; 4 °C). Supernatants were collected. Nuclear fractions were extracted using NE-PER™ Nuclear and Cytoplasmic Extraction Reagents (Thermo Fischer Scientific, Watham, MA, USA) according to the manufacturer’s instructions. Protein concentrations were determined using the Bio-Rad protein assay (Bio-Rad, Hercules, CA, USA). 

The protein extracts (20 µg from MIN6, 5 µg from islets) were resolved on 10–20% or 4–15% gradient polyacrylamide gels (Invitrogen) and were blotted onto a nitrocellulose membrane (GE Healthcare, Buckinghamshire, UK), and incubated overnight at 4 °C in Tris-buffered saline containing a 1:1000–5000 dilution of primary antibody (Supplementary Table S2). The membrane was incubated at 4 °C for 60 min in Tris-buffered saline with a 1:2000 dilution of anti-rabbit IgG and anti-mouse IgG horseradish peroxidase-conjugated secondary antibody (Cell Signaling Technology, Beverly, MA, USA). For the insulin antibody, anti-guinea pig IgG peroxidase-conjugated secondary antibody (Jackson ImmunoResearch, West Grove, PA, USA) were used. Immune complexes were visualised using an enhanced chemiluminescence substrate kit (GE Healthcare). Band intensities in the blots were quantified using NIH Image J software version 1.51s (freely available at http://rsb.info.nih.gov/ij/index.html, accessed on 1 April 2017) [47], and α-tubulin or GAPDH bands were used to adjust for loading differences.

### 4.7. Immunoprecipitation

Cells were lysed in ice-cold RIPA buffer with protease inhibitors for 10 min on ice, the lysates were then cleared by centrifuging the cells at 15,000× *g* for 10 min at 4 °C. For IP of endogenous Gsk-3α/β, 500 μg whole cell extract from each sample was incubated with Protein G Sepharose (Cell Signaling Technology) and 5 μg anti-Gsk-3α/β antibody (Santa Cruz Biotechnology, Santa Cruz, CA, USA) overnight at 4 °C with rotation. After incubation, the beads were washed thrice with RIPA buffer, followed by a final wash in 1× PBS. The immunoprecipitants were resolved by SDS-PAGE and subjected to immunoblotting. For IP of exogenously expressed proteins in HEK293, 2 μg of anti-Flag antibody (Cell Signaling Technology) was used; for HA, 2 μg of anti-HA antibody (Cell Signaling Technology) was used.

### 4.8. Phos-Tag Analysis

MIN6 cells were treated with tunicamycin in the presence or absence of Gsk-3 inhibitor for 5 h and then incubated with 25 μM MG132 for 1 h to inhibit the proteasomal degradation of ATF4. Following treatment, the cells were lysed, and protein samples were prepared. Phos-tag was performed as previously described [48]. Briefly, lysates were run on SDS-PAGE gels containing 25 μM Phos-tag acrylamide (Wako, Osaka, Japan), 50 μM MnCl_2_, and 8% acrylamide. The gels were run for 4 h at 90 V to achieve optimal separation of bands for ATF4. Prior to transfer, the gels were soaked in buffer containing 10 mM EDTA for 1 h.

### 4.9. GSK-3β Kinase Assay

Recombinant full-length human GSK-3β was purchased from Cell Signaling Technology. An in vitro GSK-3β kinase assay was performed according to the manufacturer’s instructions (Cell Signaling Technology). Briefly, 1 µg of WT or S214A mutant of ATF4, which was immuno-purified by using the Flag-beads in transfected HEK293 pretreated with GSK-3 inhibitor, was incubated with GSK-3β in kinase assay buffer (4 mM MOPS pH 7.2, 2.5 mM β-glycerophosphate, 1 mM EGTA, 0.4 mM EDTA, 4 mM MgCl_2_, 0.05 mM DTT, 40 μM BSA) containing 20 µM ATP for 20 min at 30 °C. Phosphorylation of ATF4 after SDS–PAGE was analysed with an anti-phospho-Ser antibody (Invitrogen).

### 4.10. DNA Fragmentation Assay

MIN6 cells or islets were incubated with 5 μM tunicamycin in the presence or absence of Gsk-3 inhibitors for 20 h. Cells were then lysed, and chromosomal DNA was extracted using the Quick Apoptotic DNA Ladder Detection Kit (BioVision, Milpitas, CA, USA), according to the manufacturer’s instructions. A volume of 20 μL of each sample was electrophoresed on a 1.2% agarose/EtBr gel and images were captured using ChemiDOC^TM^ XRS+ (Bio Rad).

### 4.11. Chemical Metabolic Labelling

The Click-IT AHA (l-azidohomoalaine) for the nascent protein synthesis kit (Invitrogen) was used in our study, and experiments were performed according to the manufacturer’s instructions. Briefly, insulinoma cells were seeded into 6 cm plates and allowed to grow to 80–90% confluency. Cells treated with an ER stress inducer for the indicated periods in the presence or absence of Gsk-3 inhibitor were starved with methionine-free media for 1 h prior to incubation with Click-iT AHA containing methionine-free media for 2 h. The cells were harvested and lysed. Cell lysates were labelled with biotin-conjugated alkynes, and biotin-labelled proteins were captured using streptavidin agarose resin. The biotin labelled proteins were detected by Western blotting using HRP-conjugated streptavidin for the whole cell lysates or anti-biotin antibodies for the biotin-streptavidin captured extracts.

### 4.12. Real Time PCR

Cells were washed twice with cold PBS before the addition of TRIZOL (Invitrogen), and RNA was extracted according to the manufacturer’s instructions. Islet RNA was isolated using the PicoPure RNA isolation kit (Thermo Fisher Scientific). RNA was reverse-transcribed with random hexamers using a High-Capacity cDNA Reverse Transcription Kit (Applied Biosystems, Foster City, CA, USA). Quantitative real-time PCR was performed using PowerUp SYBR Green Master Mix (Applied Biosystems) in an ABI StepOnePlus Real-Time PCR system. Relative gene expression levels were calculated by the ΔΔCt method, using Cypa (encoding *cyclophilin A*) as an internal control. Primer sequences are listed in Supplementary Table S3.

### 4.13. Firefly Luciferase Reporter Assay

Oligonucleotides containing ATF4 binding sites were annealed and sub-cloned into the pGL3-Promoter vector (BamHI-SalI) (Promega, Madison, WI, USA). MIN6 cells were cotransfected with the indicated firefly luciferase constructs and the Renilla luciferase normalisation control using Lipofectamine 2000 (Invitrogen). Lysates were collected 36 h after transfection, and firefly and Renilla luciferase activities were measured using a dual-luciferase reporter system (Promega).

### 4.14. Statics

Quantitative data are presented as the mean ± SD, unless otherwise noted. Significant differences were evaluated using either one- or two-way ANOVA, followed by Bonferroni’s post hoc test. Comparisons between two groups were performed using unpaired Student’s *t*-test. Statistical significance was set at *p* < 0.05. All statistical analyses were performed using GraphPad Prism software version 7.

## 5. Conclusions

Our work revealed that Gsk-3 activity, a negatively regulated substrate of the PI3K-Akt pathway, mediates proteasomal degradation of ATF4, and consequently alters the integrated stress response in β-cells under ER stress, leading to β-cell apoptosis. Thus, these results provided the rationale for inhibiting this enzyme to prevent the progression of β-cell failure caused by cellular stress.

## Data Availability

K.T. has full access to all data in the study. All data are available upon request.

## Supplementary Materials

Supplementary information can be downloaded at: https://www.mdpi.com/article/10.3390/ijms232113586/s1.

## References

[1] Weir G.C., Bonner-Weir S. (2013). Islet β cell mass in diabetes and how it relates to function, birth, and death. Ann. N. Y. Acad. Sci..

[2] Nolan C.J., Damm P., Prentki M. (2011). Type 2 diabetes across generations: From pathophysiology to prevention and management. Lancet.

[3] Butler A.E., Jansen J., Bonner-Weir S., Ritzel R., Rizza R.A., Butler P.C. (2003). Beta-cell deficit and increased beta-cell apoptosis in humans with type 2 diabetes. Diabetes.

[4] Poitout V., Robertson R.P. (2008). Glucolipotoxicity: Fuel excess and beta-cell dysfunction. Endocr. Rev..

[5] Prentki M., Nolan C.J. (2006). Islet beta cell failure in type 2 diabetes. J. Clin. Investig..

[6] Schröder M., Kaufman R.J. (2005). ER stress and the unfolded protein response. Mutat. Res..

[7] Eizirik D.L., Cardozo A.K., Cnop M. (2008). The role for endoplasmic reticulum stress in diabetes mellitus. Endocr. Rev..

[8] Laybutt D.R., Preston A.M., Akerfeldt M.C., Kench J.G., Busch A.K., Biankin A.V., Biden T.J. (2007). Endoplasmic reticulum stress contributes to beta cell apoptosis in type 2 diabetes. Diabetologia.

[9] Mizukami H., Takahashi K., Inaba W., Tsuboi K., Osonoi S., Yoshida T., Yagihashi S. (2014). Involvement of oxidative stress-induced DNA damage, endoplasmic reticulum stress, and autophagy deficits in the decline of β-cell mass in Japanese type 2 diabetic patients. Diabetes Care.

[10] Hetz C., Zhang K., Kaufman R.J. (2020). Mechanisms, regulation and functions of the unfolded protein response. Nat. Rev. Mol. Cell Biol..

[11] Costa-Mattioli M., Walter P. (2020). The integrated stress response: From mechanism to disease. Science.

[12] Harding H.P., Novoa I., Zhang Y., Zeng H., Wek R., Schapira M., Ron D. (2000). Regulated translation initiation controls stress-induced gene expression in mammalian cells. Mol. Cell..

[13] Vattem K.M., Wek R.C. (2004). Reinitiation involving upstream ORFs regulates ATF4 mRNA translation in mammalian cells. Proc. Natl. Acad. Sci. USA.

[14] Harding H.P., Zhang Y., Zeng H., Novoa I., Lu P.D., Calfon M., Sadri N., Yun C., Popko B., Paules R. (2003). An integrated stress response regulates amino acid metabolism and resistance to oxidative stress. Mol. Cell..

[15] Wek R.C., Cavener D.R. (2007). Translational control and the unfolded protein response. Antioxid. Redox. Signal..

[16] Zadorozhnii P.V., Pokotylo I.O., Kiselev V.V., Okhtina O.V., Kharchenko A.V. (2019). Molecular docking studies of salubrinal and its analogs as inhibitors of the GADD34:PP1 enzyme. ADMET DMPK.

[17] Yamaguchi S., Ishihara H., Yamada T., Tamura A., Usui M., Tominaga R., Munakata Y., Satake C., Katagiri H., Tashiro F. (2008). ATF4-mediated induction of 4E-BP1 contributes to pancreatic beta cell survival under endoplasmic reticulum stress. Cell Metab..

[18] Kitakaze K., Oyadomari M., Zhang J., Hamada Y., Takenouchi Y., Tsuboi K., Inagaki M., Tachikawa M., Fujitani Y., Okamoto Y. (2021). ATF4-mediated transcriptional regulation protects against β-cell loss during endoplasmic reticulum stress in a mouse model. Mol. Metab..

[19] Hurel S.J., Rochford J.J., Borthwick A.C., Wells A.M., Vandenheede J.R., Turnbull D.M., Yeaman S.J. (1996). Insulin action in cultured human myoblasts: Contribution of different signalling pathways to regulation of glycogen synthesis. Biochem. J..

[20] Beurel E., Grieco S.F., Jope R.S. (2015). Glycogen synthase kinase-3 (GSK3): Regulation, actions, and diseases. Pharmacol. Ther..

[21] Meares G.P., Jope R.S. (2007). Resolution of the nuclear localization mechanism of glycogen synthase kinase-3: Functional effects in apoptosis. J. Biol. Chem..

[22] Tanabe K., Liu Z., Patel S., Doble B.W., Li L., Cras-Méneur C., Martinez S.C., Welling C.M., White M.F., Bernal-Mizrachi E. (2008). Genetic deficiency of glycogen synthase kinase-3beta corrects diabetes in mouse models of insulin resistance. PLoS Biol..

[23] Liu Y., Tanabe K., Baronnier D., Patel S., Woodgett J., Cras-Méneur C., Permutt M.A. (2010). Conditional ablation of Gsk-3β in islet beta cells results in expanded mass and resistance to fat feeding-induced diabetes in mice. Diabetologia.

[24] Srinivasan S., Ohsugi M., Liu Z., Fatrai S., Bernal-Mizrachi E., Permutt M.A. (2005). Endoplasmic reticulum stress-induced apoptosis is partly mediated by reduced insulin signalling through phosphatidylinositol 3-kinase/Akt and increased glycogen synthase kinase-3beta in mouse insulinoma cells. Diabetes.

[25] Tanabe K., Liu Y., Hasan S.D., Martinez S.C., Cras-Méneur C., Welling C.M., Bernal-Mizrachi E., Tanizawa Y., Rhodes C.J., Zmuda E. (2011). Glucose and fatty acids synergize to promote B-cell apoptosis through activation of glycogen synthase kinase 3β independent of JNK activation. PLoS ONE.

[26] Hotamisligil G.S. (2010). Endoplasmic reticulum stress and the inflammatory basis of metabolic disease. Cell.

[27] Kim H.S., Skurk C., Thomas S.R., Bialik A., Suhara T., Kureishi Y., Birnbaum M., Keaney J.F., Walsh K. (2002). Regulation of angiogenesis by glycogen synthase kinase-3beta. J. Biol. Chem..

[28] Feng L., Li M., Hu X., Li Y., Zhu L., Chen M., Wei Q., Xu W., Zhou Q., Wang W. (2021). CK1δ stimulates ubiquitination-dependent proteasomal degradation of ATF4 to promote chemoresistance in gastric Cancer. Clin. Transl. Med..

[29] Lassot I., Ségéral E., Berlioz-Torrent C., Durand H., Groussin L., Hai T., Benarous R., Margottin-Goguet F. (2001). ATF4 degradation relies on a phosphorylation-dependent interaction with the SCF (betaTrCP) ubiquitin ligase. Mol. Cell Biol..

[30] He C.H., Gong P., Hu B., Stewart D., Choi M.E., Choi A.M., Alam J. (2001). Identification of activating transcription factor 4 (ATF4) as an Nrf2-interacting protein. Implication for heme oxygenase-1 gene regulation. J. Biol. Chem..

[31] Pakos-Zebrucka K., Koryga I., Mnich K., Ljujic M., Samali A., Gorman A.M. (2016). The integrated stress response. EMBO Rep..

[32] Mukherjee D., Bercz L.S., Torok M.A., Mace T.A. (2020). Regulation of cellular immunity by activating transcription factor 4. Immunol. Lett..

[33] Fonseca S.G., Lipson K.L., Urano F. (2007). Endoplasmic reticulum stress signalling in pancreatic beta-cells. Antioxid. Redox. Signal..

[34] Robertson H., Hayes J.D., Sutherland C. (2018). A partnership with the proteasome; the destructive nature of GSK3. Biochem. Pharmacol..

[35] Bechard M., Trost R., Singh A.M., Dalton S. (2012). Frat is a phosphatidylinositol 3-kinase/Akt-regulated determinant of glycogen synthase kinase 3β subcellular localization in pluripotent cells. Mol. Cell Biol..

[36] Novoa I., Zeng H., Harding H.P., Ron D. (2001). Feedback inhibition of the unfolded protein response by GADD34-mediated dephosphorylation of eIF2α. J. Cell Biol..

[37] Welsh G.I., Stokes C.M., Wang X., Sakaue H., Ogawa W., Kasuga M., Proud C.G. (1997). Activation of translation initiation factor eIF2B by insulin requires phosphatidyl inositol 3-kinase. FEBS Lett..

[38] Kashiwagi K., Takahashi M., Nishimoto M., Hiyama T.B., Higo T., Umehara T., Sakamoto K., Ito T., Yokoyama S. (2016). Crystal structure of eukaryotic translation initiation factor 2B. Nature.

[39] Lin S.Y., Li T.Y., Liu Q., Zhang C., Li X., Chen Y., Zhang S.M., Lian G., Liu Q., Ruan K. (2012). GSK3-TIP60-ULK1 signaling pathway links growth factor deprivation to autophagy. Science.

[40] Lin S.Y., Li T.Y., Liu Q., Zhang C., Li X., Chen Y., Zhang S.M., Lian G., Liu Q., Ruan K. (2012). Protein phosphorylation-acetylation cascade connects growth factor deprivation to autophagy. Autophagy.

[41] Nie T., Yang S., Ma H., Zhang L., Lu F., Tao K., Wang R., Yang R., Huang L., Mao Z. (2016). Regulation of ER stress-induced autophagy by GSK3β-TIP60-ULK1 pathway. Cell Death Dis..

[42] Miyazaki J., Araki K., Yamato E., Ikegami H., Asano T., Shibasaki Y., Oka Y., Yamamura K. (1990). Establishment of a pancreatic beta cell line that retains glucose-inducible insulin secretion: Special reference to expression of glucose transporter isoforms. Endocrinology.

[43] Nozaki J., Kubota H., Yoshida H., Naitoh M., Goji J., Yoshinaga T., Mori K., Koizumi A., Nagata K. (2004). The endoplasmic reticulum stress response is stimulated through the continuous activation of transcription factors ATF6 and XBP1 in Ins2+/Akita pancreatic beta cells. Genes Cells.

[44] Ueda K., Kawano J., Takeda K., Yujiri T., Tanabe K., Anno T., Akiyama M., Nozaki J., Yoshinaga T., Koizumi A. (2005). Endoplasmic reticulum stress induces Wfs1 gene expression in pancreatic beta-cells via transcriptional activation. Eur. J. Endocrinol..

[45] Hatanaka M., Tanabe K., Yanai A., Ohta Y., Kondo M., Akiyama M., Shinoda K., Oka Y., Tanizawa Y. (2011). Wolfram syndrome 1 gene (WFS1) product localizes to secretory granules and determines granule acidification in pancreatic beta-cells. Hum. Mol. Genet..

[46] Margottin F., Bour S.P., Durand H., Selig L., Benichou S., Richard V., Thomas D., Strebel K., Benarous R. (1998). A novel human WD protein, h-beta TrCp, that interacts with HIV-1 Vpu connects CD4 to the ER degradation pathway through an F-box motif. Mol. Cell..

[47] Girish V., Vijayalakshmi A. (2004). Affordable image analysis using NIH Image/ImageJ. Indian J. Cancer.

[48] Kinoshita E., Kinoshita-Kikuta E., Takiyama K., Koike T. (2006). Phosphate-binding tag, a new tool to visualize phosphorylated proteins. Mol. Cell Proteom..

