# Supplementary material for: Gsk-3-Mediated Proteasomal Degradation of ATF4 Is a Proapoptotic Mechanism in Mouse Pancreatic β-Cells
Source: Int J Mol Sci. 2022 Nov 5;23(21):13586. doi: 10.3390/ijms232113586 (PMC9657557; doi:10.3390/ijms232113586)
Supplement: Supplementary file 1 [file ijms-23-13586-s001.zip › ijms-1968791-supplementary.pdf]

**Gsk-3 mediated proteasomal degradation of ATF4 is a proapoptotic mechanism in mouse pancreatic β-cells.**

Yuko Nagao[1], Kikuko Amo-Shiinoki[1,2], Hiroko Nakabayashi[1], Masayuki Hatanaka[1] Manabu Kondo[1], Kimie Matsunaga[1], Masahiro Emoto[1], Shigeru Okuya[3], Yukio Tanizawa[1] and Katsuya Tanabe[1]

1. Division of Endocrinology, Metabolism, Haematological Sciences and Therapeutics, Yamaguchi University Graduate School of Medicine, Ube, Yamaguchi, Japan
2. Department of Diabetes Research, Yamaguchi University School of Medicine, Ube, Yamaguchi, Japan.
3. Health Administration Center, Yamaguchi University Organisation for University Education, Yamaguchi, Yamaguchi, Japan.

## Supplementary information

**Supplementary Figure 1 related to Figure 1**

**Supplementary Figure 2 related to Figure 2**

**Supplementary Figure 3 related to Figure 3**

**Supplementary Figure 4 related to Figure 4**

**Supplementary Figure 5 related to Figure 4**

**Supplementary Figure 6 related to Figure 6**

**Supplementary Table 1; shRNA sequences for mouse ATF4**

**Supplementary Table 2; List of antibodies used in this study**

**Supplementary Table 3; Primers for real-time PCR**

Supplementary Figure 1

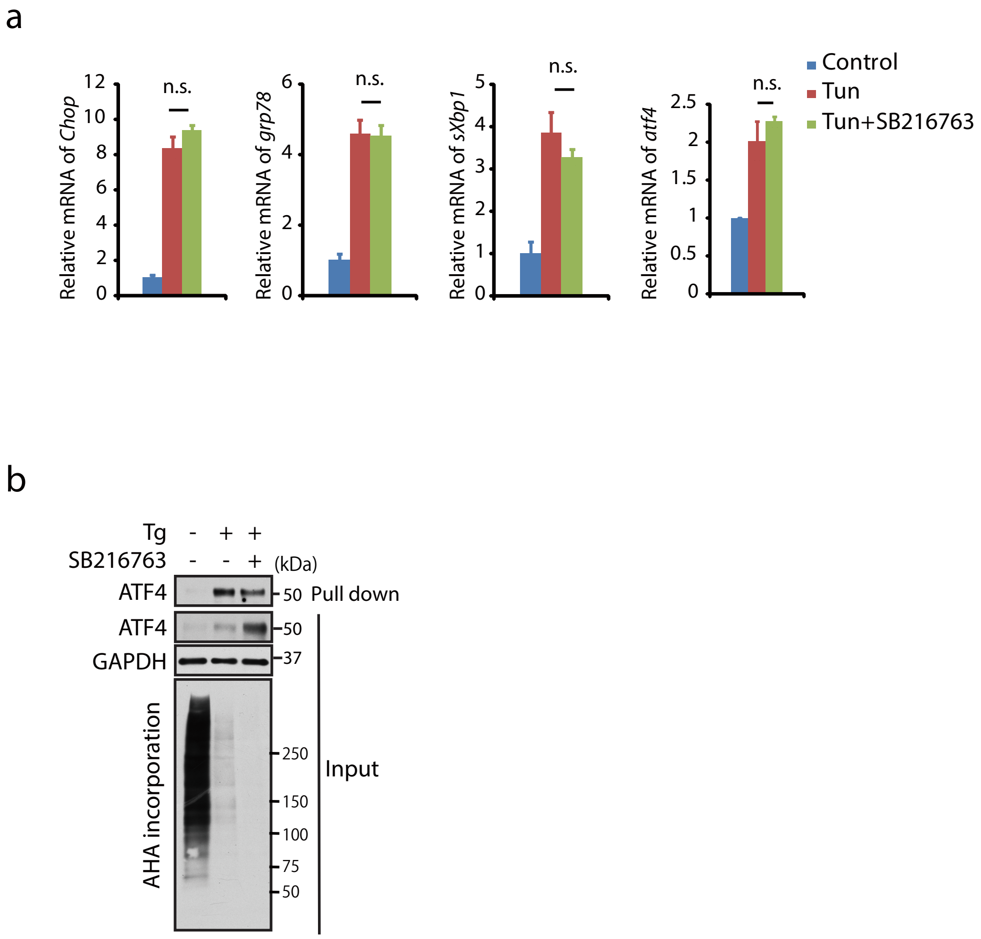


## Supplementary Figure 1

**(a)** Real time PCR on the genes involved in UPR in isolated islets incubated with tunicamycin in the presence or absence of SB216763 for 8h. Data are shown as mean ± SD. Statistical significance was assessed by one-way ANOVA followed by Bonferroni's post hoc test. **(b)** Metabolic labelling analysis in MIN6 incubated with thapsigargin (1μM) for 4h in the presence or absence of SB216763. Nascent ATF4 protein was detected in the AHA-biotin labelled protein extracts captured by streptavidin agarose resin.

Supplementary Figure 2

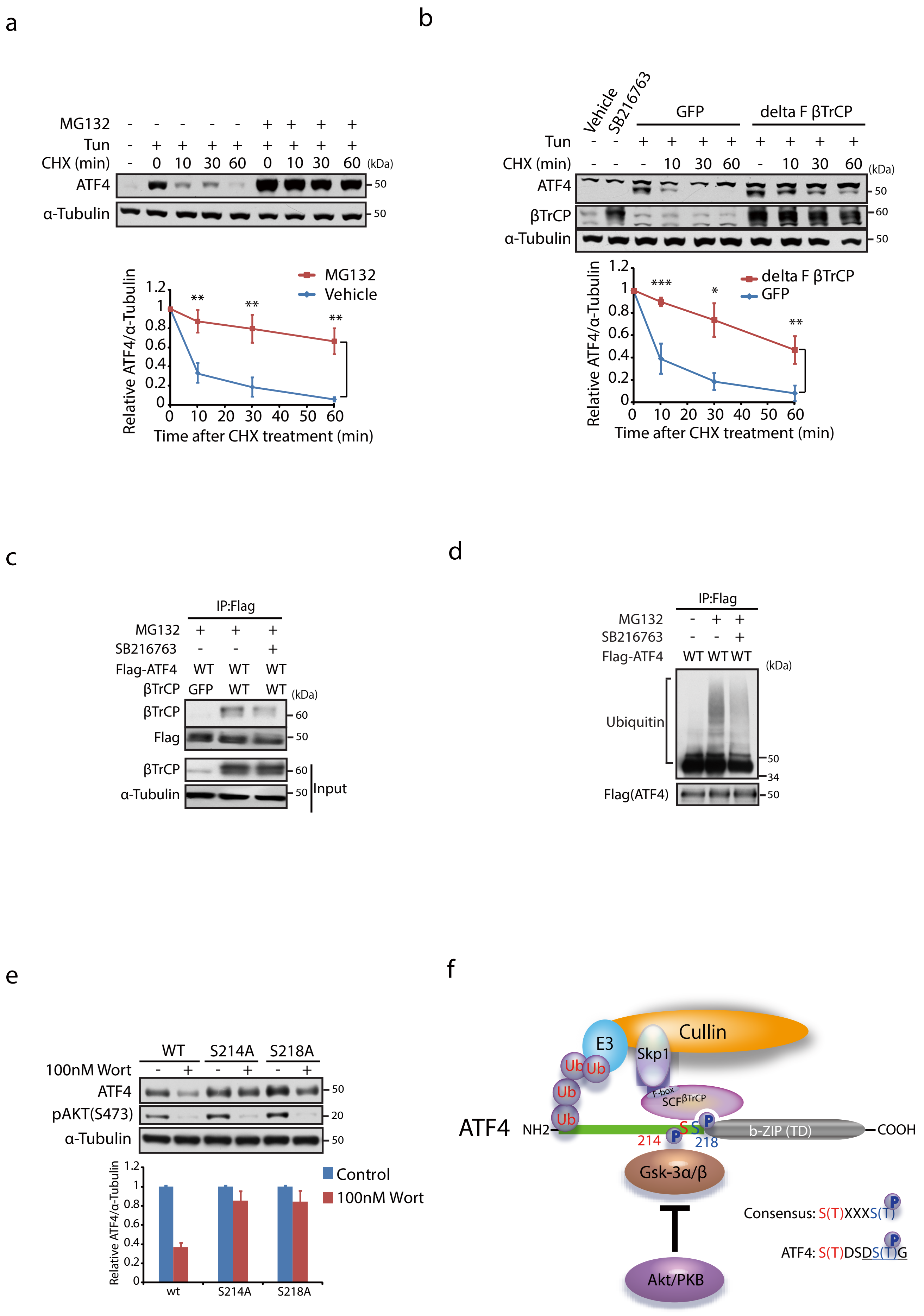

## Supplementary Figure 2

**(a)** MIN6 were incubated with tunicamycin for 4h, followed by an incubation with MG132 of 25μM for 1h. Then, CHX-chase asay was carried out and the protein lysates were extracted at indicated period. ATF4 expreession was analysed by Western blot. **(b)** CHX-chase assay of MIN6 stably expressing delta F βTrCP or GFP was carried out following a 4h incubation with tunicamysin. Protein lysates were subjected to Western blot using indicated antibodies. Graphical data represent mean ± SD for 3 independent experiments. * $p < 0.05$, ** $p < 0.01$, *** $p < 0.001$ by two-way ANOVA followed by Bonferroni's post hoc test. **(c)** HA-βTrCP was forcedly expressed together with wt Flag-ATF4 in HEK293. Immunoprecipitation was carried out using anti-Flag antibody for ATF4. βTrCP bound to ATF4 was examined by Western blot. **(d)** HEK293 transfected with pcDNA3.1-Flag-ATF4 were cotreated with MG132 and with SB216763 for 2h prior to harvest. The immunoprecipitation was performed using anti-Flag antibody for ATF4, polyubiquitin bound to ATF4 were assessed by Western blot. **(e)** An effect of wortmannin on wt, S214A and S218A mutant transiently expressed in HEK293 was analysed by Western blot. The graph represents relative change of ATF4 as mean ± SD for 2 independent experiments. **(f)** Schematic illustration representing that Gsk-3-mediated phosphorylation of ATF4-S214 facilitates a bound of βTrCP to the degron, leading to proteasomal degradation.

# Supplementary Figure 3

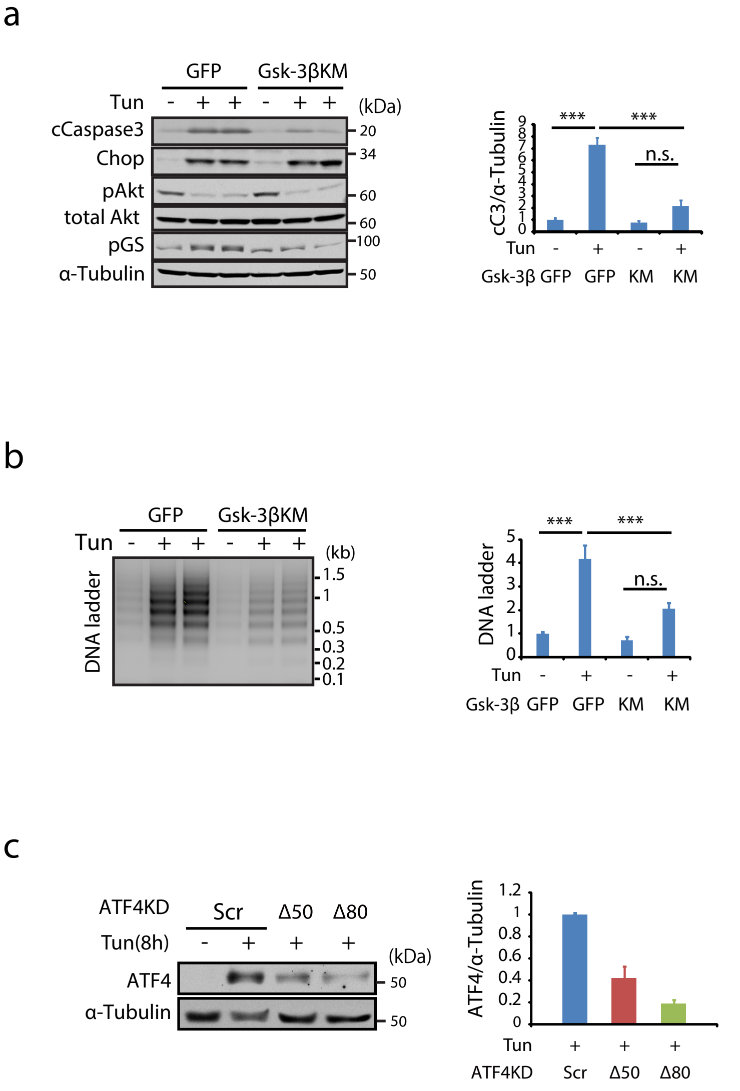


## Supplementary Figure 3

MIN6 stably expressing GFP or HA-Gsk-3KM were incubated with tunicamycin for 20h. **(a)** Representative immunoblot images obtained from the same gel and **(b)** DNA ladder assay were shown. The graphical data represent relative expression of cleaved Caspase3 and DNA ladder as mean ± SD, respectively (n=3 for each). Statistical significance was assessed by one-way ANOVA, ***$p < 0.001$. **(c)** Effects of shRNA, Δ50 and Δ80 mediated gene silencing on ATF4 protein expression in MIN6 incubated with tunicamycin for 8 hours (n=2).

# Supplementary Figure 4

a

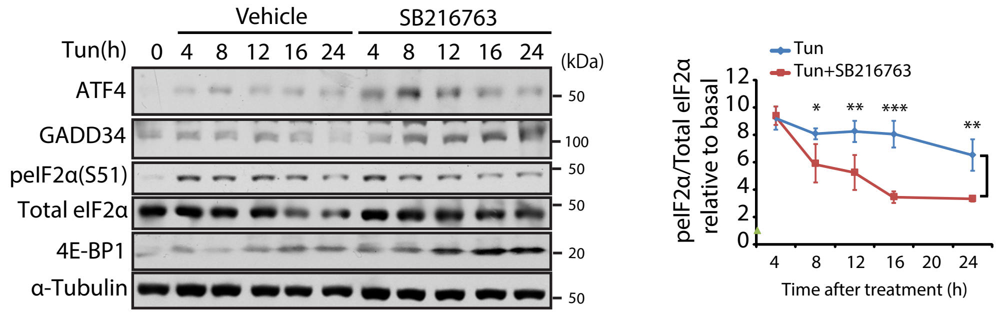


b

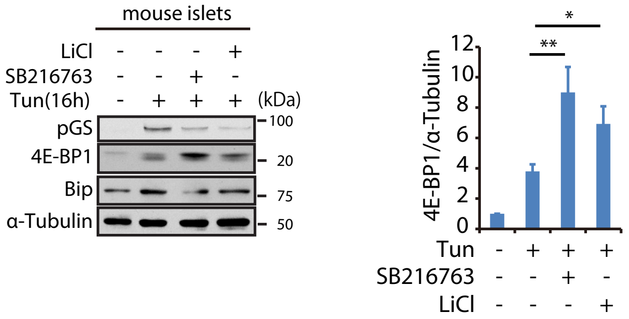


Supplementary Figure 4

**(a)** MIN6 were incubated with tunicamycin in the presence or absence of GSK-3 inhibitor for indicated periods. Protein lysates were analysed by Western blot with indicated antibodies. Representative images obtained from the same gel were shown. Time dependent changes of phosphorylation of eIF2α-S51 relative to the basal were summarised as mean ± SD (n=3). **(b)** Effects of GSK-3 inhibition on 4E-BP1 expression in isolated islets were examined by Western blot. Representative blot images obtained from the same gel were shown. Relative expression of 4E-BP1 was graphically expressed as mean ± SD (n=3). $*p < 0.05$, $**p < 0.01$, $***p < 0.001$, assessed by two-way ANOVA in **(a)** and one-way ANOVA in **(b)** followed by Bonferroni's post hoc test.

# Supplementary Figure 5

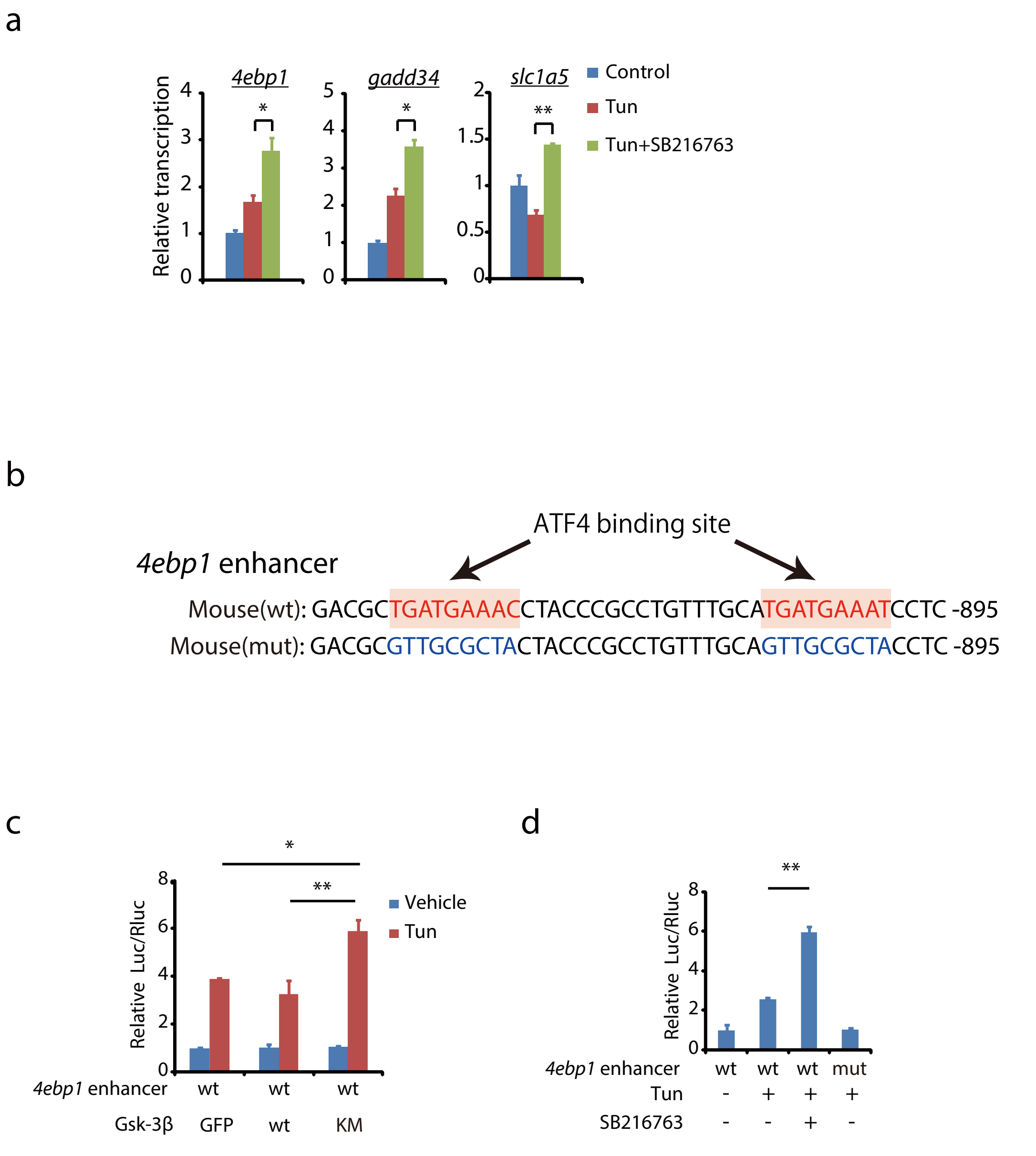


## Supplementary Figure 5

**(a)** Gene expression of ATF4 transcription targets in mouse isolated islets treated with tunicamycin in the presence or absence of SB216763 for 16h. **(b)** C/EBP: ATF composite sites in intron 1 of the mouse Eif4ebp1 gene. The number show the nucleotide position relative to the initial codon. The luciferase reporters with the SV40 promoter and an Eif4ebp1 gene segment with C/EBP: ATF composite sites or their mutants were transfected in MIN6. **(c)** The luciferase reporter activity induced by cotransfection with pcDNA3.1-GFP or pcDNA3.1-Gsk-3β (wt) or pcDNA3.1-Gsk-3βKM. **(d)** The luciferase reporter activity in the cells treated with tunicamycin in the presence or absence of SB216763. Graphical data represent mean ± SD (n = 4, respectively) Statistical significance of all graphical data were assessed by one-way ANOVA followed by Bonferroni’ s post hoc test. *p < 0.05, **p < 0.01.

Supplementary Figure 6

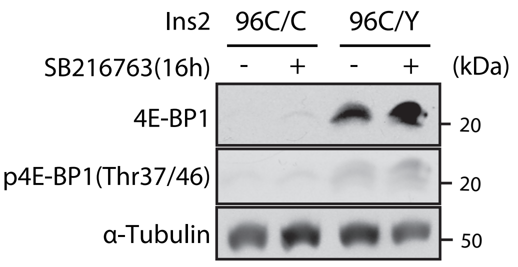


Supplementary Figure 6

The protein extracts from cells with Ins2 96C/C or those with 96C/Y incubated with either GSK-3 inhibitor or vehicle for 20 h were analysed by western blotting using indicated antibodies.

Supplementary Table 1. shRNA sequences for mouse ATF4

| Δ80 | Sequence | Position |
|---|---|---|
| target | 5’-AAACCATGCCAGATGAGCTC-3’ | 314 relative to initial ATG |
| sense | 5’-ACCATGCCAGATGAGCTCTgtgtgctgtccAGAGCTCATCTGGCATGGTTTTTT-3’ | |
| antisense | 5’-AAAAAACCATGCCAGATGAGCTCTggacagcacacAGAGCTCATCTGGCATGGT-3’ | |
| Δ50 | Sequence | Position |
| target | 5'-AAGCCTGACTCTGCTGCTTAC-3' | 577 relative to initial ATG |
| sense | 5’-GCCTGACTCTGCTGCTTACgtgtgctgtccGTAAGCAGCAGAGTCAGGCTTTTTT-3’ | |
| antisense | 5’-AAAAAAGCCTGACTCTGCTGCTTA CggacagcacacGTAAGCAGCAGAGTCAGGC-3’ | |

Supplementary Table2. List of antibodies in this study

| No | Target | Dilution | Host, MW (kDa) | Cat. No. | Company |
|---|---|---|---|---|---|
| Primary Antibodies: | | | | | |
| 1 | p-Akt (Ser473) | 1:1000 | Rabbit pAb, 60 | #9271 | Cell Signaling |
| 2 | Akt | 1:1000 | Rabbit pAb, 60 | #9272 | Cell Signaling |
| 3 | p-Gsk-3α(Ser21) | 1:1000 | Rabbit mAb, 51 | #9316 | Cell Signaling |
| 4 | p-Gsk-3β(Ser9) | 1:1000 | Rabbit mAb, 46 | #9323 | Cell Signaling |
| 5 | total Gsk-3α/β | 1:1000 | Rabbit mAb, 51,46 | #5676 | Cell Signaling |
| 6 | CHOP(GADD153) | 1:1000 | Rabbit pAb, 30 | SC-793 | Santa cruz |
| 7 | α-Tubulin | 1:5000 | Mouse mAb, 52 | #3873 | Cell Signaling |
| 8 | p-Glycogen Synthase | 1:1000 | Rabbit pAb, 85-90 | #3891 | Cell Signaling |
| 9 | Grp78/Bip | 1:1000 | Rabbit pAb, 78 | ADI-SPA-826 | Enzo |
| 10 | cleaved ATF6 | 1:1000 | Mouse mAb, 50 | IMG-273 | IMGENEX |
| 11 | ATF4 | 1:1000 | Rabbit mAb, 49 | #11815 | Cell Signaling |
| 12 | Gsk-3β | 1:1000 | Rabbit mAb, 46 | #9315 | Cell Signaling |
| 13 | Lamin A/C | 1:1000 | Rabbit pAb, 28,70 | #2032 | Cell Signaling |
| 14 | GAPDH | 1:1000 | Rabbit mAb, 37 | #2118 | Cell Signaling |
| 15 | Biotin(D5A7) | 1:1000 | Rabbit mAb, N/A | #5597 | Cell Signaling |
| 16 | HA-Tag(6E2) | 1:1000 | Mouse mAb, N/A | #2367 | Cell Signaling |
| 17 | Phosphoserine | 1:1000 | Rabbit pAb, N/A | 61-8100 | Invitrogen |
| 18 | FLAG | 1:1000 | Mouse mAb, N/A | F3165 | Sigma-Aldrich |
| 19 | Ubiquitin | 1:1000 | Rabbit pAb, N/A | #3933 | Cell Signaling |
| 20 | β-TrCP | 1:1000 | Rabbit mAb, 62 | #4394 | Cell Signaling |
| 21 | Cleaved Caspase-3(ASP175) | 1:1000 | Rabbit pAb, 17,19 | #9661 | Cell Signaling |
| 22 | p-eIF2α(Ser51) | 1:1000 | Rabbit pAb, 38 | #9721 | Cell Signaling |
| 23 | total eIF2a | 1:1000 | Rabbit pAb, 38 | #9722 | Cell Signaling |
| 24 | 4E-BP1(53H11) | 1:1000 | Rabbit mAb, 15-20 | #9644 | Cell Signaling |
| 25 | p-4E-BP1(Thr37/46) (236B4) | 1:1000 | Rabbit mAb, 15-20 | #2855 | Cell Signaling |
| 26 | GADD34(D-8) | 1:1000 | Mouse mAb, 73 | SC-46661 | Santa Cruz |
| 27 | Insulin | 1:1000 | Guinea pig mAb, 11 | A0564 | Dako |
| Secondary Antibodies: | | | | | |
| 28 | Anti-rabbit IgG, HRP-linked antibody | | | #7074 | Cell Signaling |
| 29 | Anti-mouse IgG, HRP-linked antibody | | | #7076 | Cell Signaling |
| 30 | Peroxidase AffiniPure Goat Anti-Guinea Pig IgG(H+L) | | | #106-035-003 | Jackson Immunoresearch |
| 31 | Streptavidin-HRP | | | #3999 | Cell Signaling |

p-, phosphorylated;

pAb, polyclonal antibody; mAb, monoclonal antibody

Supplementary Table3. List of primer sequences for quantitative real-time PCR

| Gene | Forward | Reverse |
|---|---|---|
| *cyclophilin A* | TGTGCCAGGGTGGTGACTTTAC | TGGGAACCGTTTGTGTTTGG |
| *insulin1* | GTGACCAGCTATAATCAGAGACCATC | AAAGCCTGGGTGGGTTTGG |
| *insulin2* | GTCAAGCAGCACCTTTGTGG | TCTGAAGGTCACCTGCTCCC |
| *atf4* | GGACAGATTGGATGTTGGAGA | AGAGGGGCAAAAAGATCACAT |
| *4ebp1* | GAGAGCTGCACAGCATTCAG | AGTCATTCCCCTGCAGTAGC |
| *bip* | TTCAGCCAATTATCAGCAAACTCT | TTTTCTGATGTATCCTATTCACCAGT |
| *chop* | CCACCACACCTGAAACGAGAA | AGGTGAAAGGCAGGGACTCA |
| *total xbp-1* | TGGCCGGGTCTGCTGAGTCCG | GTCCATGGGAAGATGTTCTGG |
| *spliced xbp-1* | CTGAGTCCGAATCAGGTGCAG | GTCCATGGGAAGATGTTCTGG |
| *gadd34* | GACCCCTCCAACTCTCCTTC | CTTCCTCAGCCTCAGCATTC |
| *slc1a5* | TCAACCATGGTCCAGCTTCT | CGGGTGCGTACCACATAATC |
| *slc7a5* | CTGCTACAGCGTAAAGGC | AACACAATGTTCCCCACGTC |
| *Slc7a1* | ATCGGTACTTCAAGCGTGGC | CCATGGCTGACTCCTTCACG |
